# The Histone Marks Signature in Exonic and Intronic Regions Is Relevant in Early Response of Tomato Genes to *Botrytis cinerea* and in miRNA Regulation

**DOI:** 10.3390/plants9030300

**Published:** 2020-03-01

**Authors:** Óscar Crespo-Salvador, Lorena Sánchez-Giménez, Mª José López-Galiano, Emma Fernández-Crespo, Loredana Schalschi, Inmaculada García-Robles, Carolina Rausell, M Dolores Real, Carmen González-Bosch

**Affiliations:** 1 Department of Biochemistry and Molecular Biology, University of Valencia, Agrochemical and Food Technology Institute (CSIC), 46980 Paterna, Valencia, Spain; oscar.crespo@uv.es (Ó.C.-S.); losangi2@alumni.uv.es (L.S.-G.); 2 Department of Genetics, University of Valencia, Burjassot, 46100 Valencia, Spain; maria.jose.lopez-galiano@uv.es (M.J.L.-G.); Inmaculada.Garcia@uv.es (I.G.-R.); Carolina.Rausell@uv.es (C.R.); Maria.Dolores.Real@uv.es (M.D.R.); 3 Plant Physiology Area, Biochemistry and Biotechnology Group, Department CAMN, University Jaume I, 12071 Castellón, Spain; ecrespo@sg.uji.es (E.F.-C.); scalschi@uji.es (L.S.)

**Keywords:** chromatin immunoprecipitation, epigenetics, histone modifications, miRNA, *Botrytis cinerea*, *Pseudomonas syringae*, tomato

## Abstract

Research into the relationship between epigenetic regulation and resistance to biotic stresses provides alternatives for plant protection and crop improvement. To unravel the mechanisms underlying tomato responses to *Botrytis cinerea*, we performed a chromatin immunoprecipitation (ChIP) analysis showing the increase in H3K9ac mark along the early induced genes *SlyDES*, *SlyDOX1*, and *SlyLoxD* encoding oxylipin-pathway enzymes, and *SlyWRKY75* coding for a transcriptional regulator of hormonal signaling. This histone mark showed a more distinct distribution than the previously studied H3K4me3. The RNAPol-ChIP analysis reflected the actual gene transcription associated with increased histone modifications. A different pattern of marks in the oxylipin-related genes against *P. syringae* supported a pathogen-specific profile, while no significant differences occurred in *SlyWRKY75*. The epigenetic regulation of *SlyWRKY75* by the intron-binding miR1127-3p was supported by the presence of *SlyWRKY75* pre-mRNA in control plants. Interestingly, mRNA was found to be accumulated in response to *B. cinerea* and *P. syringae,* while reduction in miRNA only occurred against *B. cinerea.* The intronic region presented a similar pattern of marks than the rest of the gene in both pathosystems, except for H3K4me3 in the miRNA binding site upon *B. cinerea*. We located the gene encoding Sly-miR1127-3p, which presented reduced H3K4me3 on its promoter against *B. cinerea*.

## 1. Introduction

When plants suffer the invasion of pathogens, they activate defenses to limit their expansion. These inducible responses are accompanied by extensive transcriptional reprogramming of defense-related genes [1]. Growing interest is shown in the potential roles of epigenetic control of gene expression in microbial infections [2]. This is due to the essential contributions of different epigenetic mechanisms in the immune response, including DNA and histone modifications, and post-transcriptional modulation mediated by microRNAs (miRNAs) [3]. The fact that epigenetic marks are dynamically regulated by environmental cues and relevant for transcriptional stress memory makes their study interesting from both basic and applied points of view [4]. Histone methylation and acetylation at specific residues are critical regulators of basal and primed responses. Transcriptional activation is associated with methylation of histone H3 lysine 4 (H3K4me3) and H3K36 (H3K36me3), and acetylation of H3K9, whereas gene silencing correlates with trimethylation of H3K27 (H3K27me3) [5]. Most of the available information comes from studies in *Arabidopsis thaliana* plants infected with biotrophic pathogens. Infection of Arabidopsis by *Pseudomonas syringae* pv. maculicola primed defensive genes by NPR1-dependent post-translational modifications of histone H3 at their promoter regions [6]. H3K9ac mark increased in the promoter of the SA-regulated gen *PR1* in descendants of *P. syringae* pv tomato (PstDC3000)-primed Arabidopsis plants [7]. In addition, H3K4me3 is associated with plant stress memory [8,9]. Data of epigenetic regulation in response to necrotrophs are still scarce [10]. Some evidence supports the involvement of Arabidopsis-specific histone modifying activities [11,12]. Interestingly, some toxins produced by necrotrophs can inhibit plant histone deacetylases to facilitate pathogen invasion [13]. 

Another epigenetic regulation shows relays on microRNAs (miRNAs), which are involved in many essential biological processes, including defense responses against stresses [14]. miRNAs are small non-coding RNAs, which regulate post-transcriptionally gene expression through sequence complementarity [15]. In plants, target transcripts usually contain one binding site of a single miRNA located along the mRNA. miRNA-mRNA pairing may activate transcript degradation or translational repression. The plant miRNAs are sensitive to biotic stresses in different crops. They participate in bacterial PAMP-triggered immunity (PTI) in *A. thaliana* [16], in the regulation of disease resistance genes in *Nicotiana benthamiana* [17], or in the defence of tomato plants to fungal pathogens [18,19]. Interestingly, coordination between the posttranslational modifications of histones and miRNAs genes have been demonstrated [20]. 

In recent years, we unraveled the molecular mechanisms of basal and primed tomato plant responses to necrotrophic fungi *Botrytis cinerea* [21,22]. With this information, we identified potential biomarker genes to detect the presence of *B. cinerea* in plants in early infection stages [23]. We decided to determine the epigenetic regulation of these early-responsive genes in the tomato-*B. cinerea* pathosystem, but studies in species other than *A. thaliana* were challenging [24,25,26]. In addition, information about specific histone epigenetic marks on *A. thaliana* defense-related genes against *B. cinerea* has only recently been provided [27]. This global study showed the involvement of two histone lysine methyl transferases in H3K4 and H3K36 methylation in plants infected by *B. cinerea.* Recently, we adapted a chromatin immunoprecipitation (ChIP) protocol to tomato plants infected by *B. cinerea* [28] and performed a high-throughput sequencing to analyze the microRNA profile of tomato plants under several tress conditions, including *B. cinerea* infection [29]. The analysis of histone post-translational modifications allowed to obtain a differential epigenetic signature of activating mark H3K4me3 on the promoter and body of early induced genes in the basal and hexanoic-primed responses of tomato plants to *B. cinerea* [28]. These genes were *SlyDES* (divinyl ethyl synthase), *SlyDOX1* (α-dioxygenase 1), and *SlyLoxD* (lipoxygenase D), which encode key enzymes of the oxylipins pathway. Oxylipins are lipid-derived secondary metabolites playing key roles in plant defense against pathogens [30]. Fatty acids linolenic and linoleic are the most abundant precursors of this family of molecules, whose enzymatic conversion into oxylipins initiates by either lipoxygenases (LOX) or α-dioxygenases (α-DOX). LOX plays a relevant role in plant responses to biotic and abiotic stresses, and are related mainly to LOX-derived oxylipin JA [31]. The α-DOX pathway also contributes to basal and induced resistance to insects and pathogens [32,33]. In addition, there are less-characterized oxylipins like the fungitoxic divinyl ethers colneleic acid and colnelenic acid whose formation is catalyzed by DES from 9-LOX derivatives. DES belongs to a CYP74 subfamily of cytochromes P450 not detected in mammals [34,35]. The 9-LOX pathway plays an important role in the interaction of Solanaceous plants with pathogens [36,37,38]. 

In a previous work, we also analyzed the epigenetic regulation of *SlyWRKY75*, which encodes a member of the plant transcription factor WRKY family [39]. This gene is also highly induced in tomato plants infected by *B. cinerea* [23] and could act as a transcriptional regulator of the JA pathway to defend them from biotic stress [39]. It is induced in the defense response to other necrotrophs in Arabidopsis plants [40,41], and in strawberry [42]. In *A. thaliana*, AtWRKY75 is a transcriptional regulator of the SA- and JA/ET-dependent defense signaling pathways by acting as a negative and positive regulator, respectively [40]. AtWRKY75 is also a positive regulator of leaf senescence by activating SA production and H_2_O_2_ accumulation in a tripartite amplification loop [43].

In our study, *SlyWRKY75* transcript accumulation in response to *B. cinerea* correlated with H3K4me3 increase throughout the gene but also with Sly-miR1127-3p miRNA repression [39]. This putative epigenetic regulator of *SlyWRKY75* gene expression is predicted to bind the intronic region of the genomic sequence. This is a novel post-transcriptional regulation targeting intron-containing pre-mRNAs in the nucleus [44].

We herein determined the presence of histone H3 acetylated on lysine 9 (H3K9ac), an epigenetic mark also associated with transcriptional activation in response to environmental stresses [45] in *SlyDES*, Sly*DOX1, SlyLoxD*, and *SlyWRKY75* genes in the same previously analyzed infected tomato plants [28]. To determine whether the histone marks on the studied genes were stress-specific, we analyzed the expression of these genes and the presence of both epigenetics marks H3K4me3 and H3K9ac on tomato plants inoculated with the hemibiotroph *P. syringae*. We also detected RNA polymerase II (RNAPII) by ChIP within the coding region of these stress-responsive genes to measure actual transcription. RNAPol-ChIP allowed us to isolate specific RNA Pol II–DNA complexes by using a given RNA Pol II (RNAPII) antibody [46].

We also searched whether chromatin modifications cooperated with Sly-miR1127-3p as epigenetic factors to modulate *SlyWRKY75* gene expression. For this purpose, in infected plants, we determined changes of H3K4me3 and H3k9ac marks, and RNAPII in the *SlyWRKY75* intronic region where this miRNA has been predicted to bind. We also analyzed the presence of histone marks and RNAPII on the structural gene of Sly-miR1127-3p miRNA in plants infected with either pathogen.

The obtained results provided novelty data about the epigenetic regulation of tomato biotic stress-responsive genes. This information might also help to select specific biomarkers for the early detection of devastating pathogens in relevant crops.

## 2. Results

### 2.1. Histone Mark H3K9ac on SlyDES, SlyDOX1, SlyLoxD, and SlyWRKY75 Genes in Tomato Plants’ Early Response to B. cinerea

**Figure 1:**
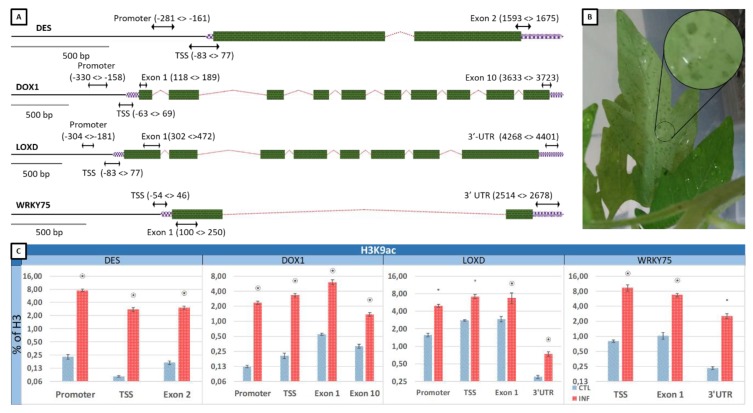
Profile of H3K9ac in genes *SlyDES*, *SlyDOX1*, *SlyLoxD* and *SlyWRKY75* in the *B. cinerea*-infected *S. lycopersicum* plants. (**A**) Schematic diagram of genes *SlyDES, SlyDOX1, SlyLOXD, and SlyWRKY75* with the promoter region (left), the 5’-UTR and 3’-UTR regions (small purple boxes at both ends), exons (big green boxes inside) and introns (upwardly angled lines) analyzed by chromatin immunoprecipitation and real-time qPCR (ChIP-qPCR). (**B**) Picture of *S. lycopersicum* leaflets under *B. cinerea* infection. (**C**) A representative experiment showing the ChIP-qPCR analysis of the amount of H3K9ac in the control and infected plants of *S. lycopersicum* at 24 hpi in different regions of the above-mentioned genes. The ChIP results are presented as percentages of chromatin immunoprecipitated with the anti-H3 antibody. Data are shown as log_2_-fold changes, error bars indicate s.e.m. (*n* = 3). The statistically significant results among technical replicates in the representative experiment shown are indicated by an asterisk (*). A star is included (⍟) if results are statistically significant in three experiments. Blue bars: control (CTL); Red bars: infected (INF).

Genes *SlyDES*, *SlyDOX1* and *SlyLoxD* that encode key oxylipin pathway enzymes and *SlyWRKY75* that codes for a stress-responsive transcription factor were highly induced in response to *B. cinerea* at 24 hpi. We previously demonstrated by ChIP the increase of H3K4me3 along these genes associated with their induction in this pathosystem (Tables S1 and S2) [28,39]. Here, we extended the analysis by exploring the presence of epigenetic mark H3K9ac on the promoter and inside the body regions of these genes which, together with H3K4me3, correlated with active transcription [3]. H3K9 acetylation did not significantly change in constitutively expressed genes *ACT2* and *EIF4* upon *B. cinerea* (Table S1). However, H3K9ac increased throughout the analyzed regions in all the pathogen-induced genes associated with gene activation at 24 hpi (Figure 1C, Table S2). The rise in this histone modification was greater than that previously determined for H3K4me3 in most of the analyzed regions. In addition, it showed a different profile with a higher increase in the promoter and TSS, while H3K4me3 was more homogeneously increased throughout the gene [28] (Table S3). A detailed analysis showed the greatest enrichment of H3K9ac along the most induced gene *SlyDES* (Figure 1C). The highly expressed *SlyDOX1* and *SlyWRKY75* genes presented a similar profile of H3K9ac distribution, but with a less marked increase. As for the least induced gene *SlyLoxD*, although H3K9 acetylation increased throughout the gene, the result was statistically significant only in the three biological replicates in the exon 1 and 3´-UTR regions (Figure 1C, Table S1).

These results demonstrated that early gene induction upon *B. cinerea* infection was associated with an increase in the H3K9ac mark along genes.

### 2.2. Histone Marks H3K9ac and H3K4me3 on SlyDES, SlyDOX1, SlyLoxD, and SlyWRKY75 Genes in Tomato Plants Infected by P. syringae

**Figure 2:**
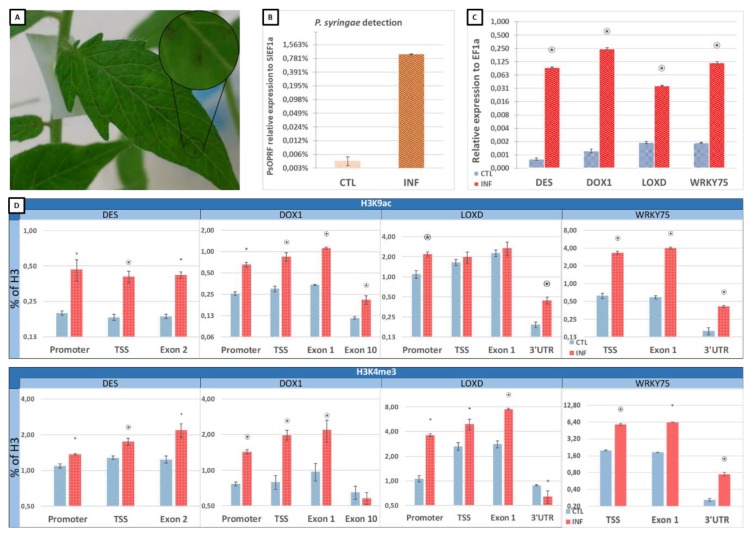
Profile of H3K9ac and H3K4me3 in genes *SlyDES, SlyDOX1, SlyLoxD, and SlyWRKY75* in the *P. syringae*-infected *S. lycopersicum* plants. (**A**) Picture of *S. lycopersicum* leaflets under *P. syringae* infection. (**B**) Determination of the presence of *P. syringae* on *S. lycopersicum* plants. Data represent the qPCR amplification of the *P. syringae* outer membrane protein F (*PsOPRF*) cDNA in relation to the *S. lycopersicum* elongation factor 1-alpha (Sl*EF1a*) cDNA in the control and infected plants. (**C**) Relative expression of the above-mentioned genes in the control and infected plants at 48 hpi. *SlyEF1a* was used as an internal standard. (**D**) A representative experiment showing the ChIP-qPCR analysis of the amount of H3K9ac and H3K4me3 in the control and infected plants of *S. lycopersicum* at 48 hpi in different regions of the above-mentioned genes. The ChIP results are presented as percentages of chromatin immunoprecipitated with the anti-H3 antibody. In all three graphics, data are shown as log_2_-fold changes, error bars indicate s.e.m. (*n* = 3). The statistically significant results among technical replicates in the representative experiment shown are indicated by an asterisk (*). A star is included (⍟) if results are statistically significant in three experiments. Blue or light brown bars: control (CTL); Red or brown bars: infected (INF).

To establish the stress-specificity of the epigenetic marks in the genes induced in response to the necrotroph *B. cinerea,* we analyzed H3K4me3 and H3K9ac on tomato plants infected by the hemibiotroph *P. syringae* pv tomato (DC3000) (Table S4). The studied genes were induced in response to this pathogen at 48 hpi (Figure 2C, Table S2). The ChIP analysis showed the rise of both epigenetic marks along the studied genes, which was not statistically significant in the three biological replicates for some regions of the genes (Figure 2D, Table S5). The increase in H3K4me3 and H3K9ac was significantly less than that observed upon *B. cinerea* along *SlyDES* and *SlyDOX1* (Table S3). However, *SlyWRKY75* showed a significant increase in both marks along the gene with more H3K9ac in exon 1 than in response to *B. cinerea* (Figure 2D, Tables S3 and S5). 

Therefore, the ChIP analysis demonstrated a different profile of epigenetic marks in response to pathogens with distinct infection strategies that seems gene dependent.

### 2.3. Presence of RNAPII in SlyDES, SlyDOX1, SlyLoxD, and SlyWRKY75 in Response to B. cinerea and P. syringae

**Figure 3:**
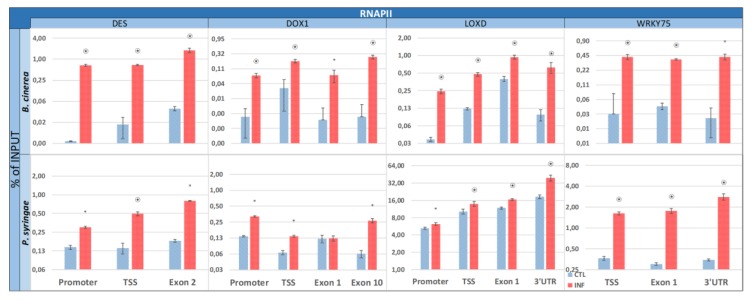
Profile of RNAPII in genes *SlyDES, SlyDOX1, SlyLoxD, and SlyWRKY75* in the *B. cinerea*- and *P. syringae*-infected *S. lycopersicum* plants. A representative experiment showing the ChIP-qPCR analysis of the amount of RNAPII in the control and infected plants of *S. lycopersicum* at 24 hpi and 48 hpi (*B. cinerea* and *P. syringae*, respectively) in different regions of the above-mentioned genes. The ChIP results are presented as percentages of input DNA. Data are shown as log_2_-fold changes, error bars indicate s.e.m. (*n* = 3). The statistically significant results among technical replicates in the representative experiment shown are indicated by an asterisk (*). A star is included (⍟) if results are statistically significant in three experiments. Blue bars: control (CTL); Red bars: infected (INF).

The detection of RNAPII within the coding region of genes by RNApol-ChIP, in combination with quantitative PCR, is a method used to accurately measure transcriptional rates [46]. In *SlyDES*, *SlyDOX1*, and *SlyLoxD* genes, the presence of RNAPII throughout the gene body increased in the plants infected by *B. cinerea*, which confirmed their transcriptional induction associated with the gain in epigenetic marks (Figure 3, Table S1). However, in *P. syringae* infected plants, RNAPII was less enriched in these oxylipin-related genes than in *B. cinerea*, despite the high induction they show in this pathosystem (Table S3). This suggests that additional regulatory mechanisms may regulate transcript accumulation in response to *P. syringae*. The fact that these genes also showed a lower increase in H3K9ac and H3K4me3 in response to this pathogen points to a connection between epigenetic marks and RNAPII presence. A different situation was observed in *SlyWRKY75*, in which the presence of RNAPII was enriched in response to both pathogens at a similar level, which seemed to coincide with increased histone modifications and gene induction in both pathosystems (Figure 3, Tables S3 and S5).

The obtained results supported the early transcriptional induction of defensive genes in response to *B. cinerea*, while additional regulatory mechanisms may be involved against *P. syringae*.

### 2.4. Epigenetic Marks H3K9ac and H3K4me3 and the Presence of RNAPII at the SlyWRKY75 Intron in Response to B. cinerea or P. syringae

**Figure 4:**
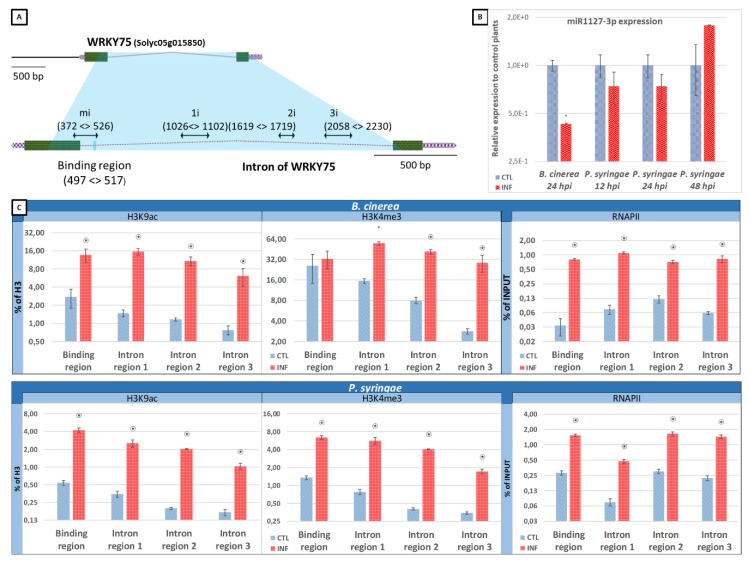
Profile of H3K9ac, H3K4me3 and RNAPII in the *WRKY75* intron in the *B. cinerea*- and *P. syringae*-infected *S. lycopersicum* plants. (**A**) Amplified schematic diagram of the *WRKY75* intron with the 5’-UTR and 3’-UTR regions (small purple boxes at both ends), exons (big green boxes inside) and the intron (upwardly angled lines). Three regions, plus the miR1127-3p binding one, were analyzed by chromatin immunoprecipitation and real-time qPCR (ChIP-qPCR). (**B**) miR1127-3p expression in the control and infected plants at 12, 24, and 48 hpi (for *P. syringae*, only 24 hpi for *B. cinerea*). Data represent the mean of three biological replicates. Asterisk indicates that differences between means of control and infected plants were statistically significant. (**C**) A representative experiment showing the ChIP-qPCR analysis of the amount of H3K9ac, H3K4me3 and RNAPII in the control and infected plants of *S. lycopersicum* at 24 hpi and 48 hpi (*B. cinerea* and *P. syringae* respectively) in different intron regions. The ChIP results are presented in the histone marks graphics as percentages of chromatin immunoprecipitated with the anti-H3 antibody, and in the RNAPII ones as percentages of input DNA. Data are shown as log_2_-fold changes, error bars indicate s.e.m. (*n* = 3). The statistically significant results among technical replicates in the representative experiment shown are indicated by an asterisk (*). A star is included (⍟) if results are statistically significant in three experiments. Blue bars: control (CTL); Red bars: infected (INF).

A previous study identified putative epigenetic regulator Sly-miR1127-3p, whose predicted binding site is located in the *SlyWRKY75* intronic region [39]. Therefore, this miRNA could regulate gene expression differently to those described as canonical post-transcriptional mechanisms [47]. We confirmed that the *SlyWRKY75* transcript increased and Sly-miR1127-3p reduced at 24 hpi of *B. cinerea*, while the miRNA level did not change when this gene was induced in response to *P. syringae* at 48 hpi (Figure 4A). This supported a more specific regulation of *SlyWRKY75* by Sly-miR1127-3p in response to *B. cinerea*, as previously reported [39]. In order to rule out an earlier differential expression of miRNA in response to *P. syringae*, we determined Sly-miR1127-3p miRNA and *SlyWRKY75* accumulation at 12 h and 24 h after *P. syringae* inoculation. The miRNA did not significantly change at earlier times upon infection, which supported a different *SlyWRKY75* regulation in response to the hemibiotropic bacterium (Figure 4B, Table S6).

By ChIPqPCR, we analyzed histone marks H3K9ac and H3K4me3 in the *SlyWRKY75* intron in the tomato plants infected with *B. cinerea* or *P. syringae.* We searched for four different locations inside the long intron, including the proposed miRNA binding site. In response to both pathogens, we observed a significant increase in the epigenetic marks along the intron, like previously observed in the rest of the gene, showing more H3K9ac in response to *P. syringae* than to *B. cinerea* (Figure 4B, Table S3). Interestingly, in its 5’ regions, including the miRNA binding site, H3K4me3 increased upon *P. syringae*, while in response to *B. cinerea*, no significant changes were observed among replicates (Figure 4B, Table S3). The presence of RNAPII increased along the intron in both pathosystems to correlate with the actual induced transcription (Figure 4C, Tables S1, S3 and S5).

These results provided evidence for the increase of activating marks in the intronic region of a plant gene induced in response to pathogens. They also showed changes in the profile of epigenetic marks at the miRNA binding site of *SlyWRKY75* intron in response to *B. cinerea* or *P. syringae*. 

### 2.5. Analysis of SlyWRKY75 Transcript Processing in Response to B. cinerea or P. syringae 

**Figure 5:**
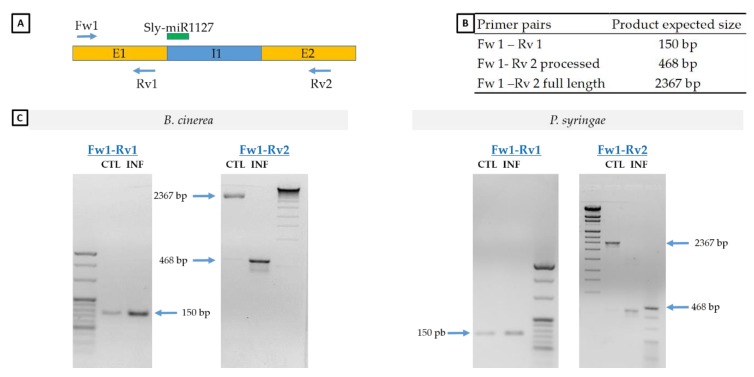
RT-PCR amplification of *SlyWRKY75* mRNA. (**A**) Schematic diagram of SlyWRKY75 gene structure. Green bar indicates sly-miR1127-binding site in *SlyWRKY75* intron 1. Arrows indicate the annealing positions of primer pair Fw1 and Rv1 in exon 1 and Rv2 in exon 2. (**B**) Table showing the different amplicons sizes. (**C**) RT-PCR analysis of SlyWRKY75 mRNA in control tomato plants and tomato plants 24 h after infection by *P. syringae* or *B. cinerea* using primer pair Fw1-Rv1 or Fw1-Rv2. For each sample, three biological replicates were pooled and analysed.

To further assess the involvement of Sly-miR1127-3p in the epigenetic regulation of *SlWRKY75* in response to pathogens, we analyzed *SlWRKY75* mRNA in plants challenged with *B. cinerea* or *P. syringae*. We designed two pairs of primers to amplify SlWRKY75 mRNA fragments in small RNA samples isolated from total RNA of control tomato plants and tomato plants 24 h after *B. cinerea* inoculation or *P. syringae* inoculation (Table S8). Figure 5A shows the annealing positions of both pairs of PCR primers. The primer pair Fw1 and Rv1 anneals to sequences flanking the predicted Sly-miR1127-3p-binding intronic region, yielding a 150 bp SlyWRKY75 amplification product. The primer pair Fw1 and Rv2, anneals to sequences within a SlyWRKY75 mRNA region downstream of the intron yielding a 2367 bp when the SlyWRKY75 mRNA contains the intronic sequence or a 468 bp SlyWRKY75 amplification product when the mRNA has been processed (Figure 5B). Figure 5C shows the results obtained in the RT-PCR amplifications using the two pairs of primers. Higher amounts of amplification products were obtained using primers Fw1 and Rv1 in tomato plants infected with *B. cinerea* or *P. syringae* compared to control plants as expected for this pathogen-induced gene. Interestingly, a 2367 bp product corresponding to SlyWRKY75 pre-mRNA was amplified using primers Fw1 and Rv2 in control plants, while a 468 bp product of processed mRNA was observed in plants infected with *B. cinerea* or *P. syringae*.

These results support post-transcriptional regulation of *SlyWRKY75* by Sly-miR1127-3p in response to pathogen infection. However, the pathogen-induced *SlyWRKY75* mRNA only correlated with an opposite pattern of expression of Sly-miR1127-3p in plants infected by *B. cinerea*. In addition, as previously shown, the miRNA binding site presented different epigenetic marks in response to each pathogen. Therefore, we hypothesize that additional factors may contribute to modulate gene silencing in response to *P. syringae.*

### 2.6. Epigenetic Marks H3K9ac and H3K4me3 and Presence of RNAPII in the Sly-miR1127-3p Structural Gene in Response to B. cinerea and P. syringae

**Figure 6:**
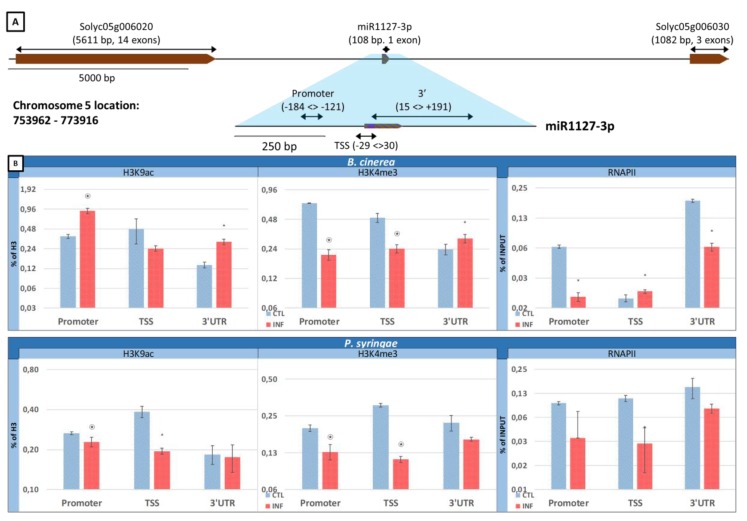
Profile of H3K9ac, H3K4me3 and RNAPII in miR1127-3p in the *B. cinerea*- and *P. syringae*-infected *S. lycopersicum* plants. (**A**) Schematic diagram of a 20354 bp chromosome 5 region containing miR1127-3p at the center with Solyc05g006020 to its 5’ side (right) and Solyc05g006030 to its 3’ (left), both represented as brown boxes. Below the zoomed-in 843 bp area containing the miR1127-3p gene, the promoter, and the TSS and 3’ regions are analyzed by chromatin immunoprecipitation and real-time qPCR (ChIP-qPCR). (**B**) A representative experiment showing the ChIP-qPCR analysis of the amount of H3K9ac, H3K4me3 and RNAPII in the control/infected plants of *S. lycopersicum* at 24 hpi and 48 hpi (*B. cinerea* and *P. syringae*, respectively) in the afore mentioned gene regions. The ChIP results are presented in the histone marks graphics as percentages of chromatin immunoprecipitated with the anti-H3 antibody, and in the RNAPII ones as percentages of input DNA. Data are shown as log_2_-fold changes, error bars indicate s.e.m. (*n* = 3). The statistically significant results among technical replicates in the representative experiment shown are indicated by an asterisk (*). A star is included (⍟) if results are statistically significant in the three experiments. Blue bars: control (CTL); Red bars: infected (INF).

We identified the gene encoding Sly-miR1127-3p miRNA, which consisted of 108 bp, including the 21 bp binding sequence to the mRNA in its 5’ end. This gene is located in a 13,261 bp intergenic region at chromosome 5is at 3627 bp from the 5’ end of a gene coding for a nucleobase-ascorbate transporter 7 (Solyc05g006020), and at 9923 bp from the 3’ end of a gene encoding for an uncharacterized protein LOC101263113 (Solyc05g006030) (Figure 6A). We designed primers for its promoter, TSS and 3´ regions, to analyze the presence of epigenetic marks in plants infected by *B. cinerea* or *P. syringae* relative to the control plants. The level of H3K9ac and H3K4me3 was significantly low throughout the gene, with a reduction in H3K4me3 in its promoter and TSS being the most significant change upon *B. cinerea*, while no conclusive results were obtained in response to *P. syringae* (Figure 6B, Tables S1, S3 and S5). A significant reduction in RNAPII was observed among replicates in both pathosystems.

These results provided evidence for changes in the epigenetic marks of a tomato gene encoding a pre-miRNA in response to pathogens.

## 3. Discussion 

Epigenetic control of gene expression is important for the transcriptional reprogramming of plant responses to stresses and may constitute molecular memory for increased protection, even across generations [2]. We are interested in establishing the epigenetic regulation of tomato responses to *B. cinerea*, which has a broad-host spectrum and requires a complex network of genes and pathways for full resistance [48]. Despite being a model for necrotrophs, information on specific histone marks on defensive genes against this important phytopathogen is scarce [10]. We previously showed the increase of the histone modification H3K4me3 in *SlyDES*, *SlyDOX1*, *SlyLoxD*, and *SlyWRKY75* genes 24h after *B. cinerea* infection [28,39]. These genes were selected due to their early induction in the basal and primed tomato defences against this fungus and by their key role in the plant response to biotic stresses [23]. Our experiments herein demonstrated that the early induction of these genes in response to *B. cinerea* is associated with enhanced epigenetic mark H3K9ac on their promoter and gene body. Thus, as for H3K4me3, this supports the notion that H3 modifications occur throughout plant genes [28,49]. 

The enrichment of H3K9ac was higher than that observed for H3K4me3 and mainly accumulated at the 5’ part of the genes, while H3K4me3 was more homogeneous along them (Table 1) [28]. This profile contrasts with that observed along defense genes *AtPR1* and *AtCYP71A13* in *A. thaliana* plants, whose induction upon *B. cinerea* infection was associated with a similar enrichment of both H3K9ac and H3K4me3 along the genes, which was less marked in the promoter [28]. In mammalian cells, H3K4me3 can promote transcription initiation, while H3K9ac can function downstream by recruiting proteins by stimulating transition to the elongation step [50]. The authors suggest that an ordered histone code can mediate the regulation of the transcription cycle. Our results seem to support this intriguing idea with a different profile in plants, which will require further research. We also determined the RNAPII location in the body of genes to study the interplay between transcription and chromatin modifications [46]. There are data associating specific histone marks on stress-responsive genes with their level of expression [5]. The RNApol-ChIP analysis showed that RNAPII rose along all the induced genes in response to *B. cinerea* supporting their transcriptional activation, associated with the increased H3K9ac and HK4me3 marks. However, it is difficult to assess the correlation between both facts. Modifications at specific histone residues are linked to chromatin reconfiguration in response to biotic stresses and are involved in priming defenses by altering the transcription of defense-related genes [5,6]. However, these marks may correlate with, or be a consequence of, transcriptional reprogramming that takes place upon a pathogen challenge [9]. In fact, dehydration stress is associated to elevated H3K4me3 at the response genes, while this mark increased before actual transcription by an unclear mechanism [4].

The analysis of these histone modifications in tomato plants inoculated with the hemibiotroph *P. syringae* showed a completely different pattern of both histone marks in *SlyDES*, *SlyDOX1*, and *SlyLoxD* genes. We observed a slight increase in H3K9ac and H3K4me3 along these oxylipin-related genes, which supports a pathogen-specific profile of epigenetic marks. The presence of RNAPII did not significantly increase in the highly induced *SlyDES* and *SlyDOX1* upon *P. syringae*. However, the induction of *SlyLoxD* in this pathosystem was associated with increased RNAPII presence in the gene body. Therefore, the obtained results might be explained by different transcriptional and epigenetic regulation in response to *B. cinerea* or *P. syringae* leading to changes in transcript stability that deserve further research. Regarding *SlyWRKY75*, the increase in both epigenetic modifications along the gene was even higher than in response to *B. cinerea*. Interestingly, upon *P. syringae* infection, *SlyWRKY75* showed a significant increase in RNAPII binding along the gene, supporting its transcriptional induction in this pathosystem too. In this case, the polymerase presence was higher than that observed in response to *B. cinerea*, correlating with the higher accumulation of *SlyWRKY75* transcript and H3K9ac mark in response to the bacterial infection. The similar increase of H3K9ac and H3K4me3 along this gene in these two pathosystems suggests that this epigenetic profile is associated with a general response to biotic stresses. This is particularly interesting because this transcription factor might be a regulator of the JA pathway in tomato, which is a key signaling in plant defense against pathogens [39]. To demonstrate the functional correlation between these histone modifications and the transcriptional activation of these defensive genes, it would be necessary to perform a direct phenotypic characterization of loss-of-function mutants defective in enzymes involved in histone modification. This is particularly challenging in plant species different to *A. thaliana* [24].

**Scheme 1:**
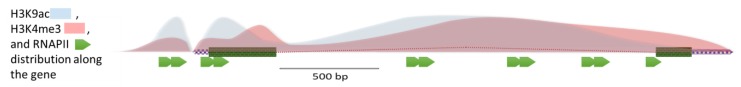
Schematic distribution of H3K9ac, H3K4me3, and RNAPII along *SlyWRKY75* in the *B. cinerea*-infected *S. lycopersicum* plants based on the obtained results. The promoter region is on the left side of the gene. 5’-UTR and 3’-UTR regions are represented as small purple boxes at both ends of the gene body; exons are big green boxes inside the gene and intron is the upwardly angled line. H3K9ac enrichment corresponds to the light blue curve, while H3K4me3 is depicted by the pink one. RNAPII is shown below the gene as a green arrow.

Recently, we identified Sly-miR1127-3p miRNA as a putative regulator of *Sly-WRKY75*, which is predicted to bind the intronic region of its genomic sequence [39]. In plants, most target mRNAs contain only one single miRNA-complementary site and, unlike animal miRNA targets, this site in plants can exist anywhere along the target mRNA, rather than at the 3´-UTR [44]. In fact, Meng et al. (2013) proposed a novel gene regulation by plant miRNAs targeting intron-containing pre-mRNAs in the nucleus. Here, we found that in response to *B. cinerea*, Sly-miR1127-3p reduction correlated with increased *SlyWRKY75* transcript accumulation, while no significant changes in miRNA occurred in response to *P. syringae* despite of *SlyWRKY75* induction. The mRNA analysis further supported the novel post-transcriptional regulation of *SlyWRKY75* targeting intron-containing pre-mRNAs. In fact, healthy control tomato plants contained *SlyWRKY75* pre-mRNA, while upon infection with either *B. cinerea* or *P. syringae* accumulated mature mRNA. This is interesting because *SlyWRKY75* transcriptional induction only correlated with reduction in the regulatory miRNA in response to *B. cinerea.* This might reflect a different epigenetic regulation of this gene in both pathosystems being plants less sensitive to miRNA steady-state concentration in response to *P. syringae*. The ChIP analysis showed a similar distribution of H3K9ac and H3K4me3 marks in the intron to that observed in the rest of *SlyWRKY75* gene in both pathosystems, except for its 5´ half, including the miRNA binding site in response to *B. cinerea*, for which no significant changes in H3K4me3 were noted. There are previous reports supporting additional factors, including binding sequence structure and surroundings, or RNA binding proteins modulate silencing efficiency [15]. Although there are evidences of the influence of chromatin structure of target genes on miRNA-regulation, no correlation has been established with specific histone modifications [51]. We hypothesize that the different profile of H3K4me3 we found in the miRNA binding site in response to each pathogen could contribute to determining the sensitivity to this epigenetic regulator. The presence of RNAPII greatly increased along the gene, including all the intronic regions for either pathogen, which reflected actual induced transcription (Scheme 1). 

These results are particularly relevant because introns are involved in initiating and enhancing gene expression by a mechanism known as intron-mediated enhancement (IME) which, in plants, is largely unknown [52]. Stimulating introns constitutes a type of downstream regulatory element for the genes transcribed by RNAPII by increasing mRNA production and contributing to local chromatin conditions that favor initiation. The distribution of active histone modifications like H3K9ac and H3K4me3 in introns could influence transcription initiation in Arabidopsis [53]. Some reports reveal that the first intron of genes is generally the longest one and contains more conserved sites and regulatory chromatin marks [54]. The density of epigenetic signals is higher near TSS than in distant regions, and their distribution seems related to gene function and its level of expression. In fact, highly expressed genes exhibit a higher density of marks than genes with a low expression. Our results in this work provide data supporting the role of intron sequences in the epigenetic regulation of plant defense genes, thus encouraging future studies in this fascinating field.

Finally, to study in-depth *SlyWRKY75* epigenetic regulation, we located the structural Sly-miR1127-3p gene in an intergenic region of the tomato genome (Figure 6A). This agrees with the fact that plant miRNAs transcribe mostly from independent, nonprotein-coding loci in the intergenic regions of the genome, although intronic miRNAs and transposable elements are other sources of miRNA formation in plants [15]. Throughout this gene, the level of both activating marks H3K9ac and H3K4me3, as well as RNAPII, was very low. We were able to detect only changes in response to *B. cinerea*, which consisted in a significant reduction of H3K4me3 in TSS and the promoter, with the latter associated with an increase in H3K9ac. These results show different histone modifications on the gene encoding Sly-miR1127-3p in response to *B. cinerea* or *P. syringae*, although these cannot be directly correlated with changes in the miRNA accumulation because no significant changes in RNAPII presence were detected. 

In conclusion, our data provide knowledge about the epigenetic regulation of genes involved in plant response to *B. cinerea* and *P. syringae* in crops other than *A. thaliana*, such as *S. lycopersicum*, which is still limited and challenging. Our research deeps into the relationship between epigenetics and biotic stresses that provides information useful to prevent and early detect pathogens in economically relevant crops. The identification of key loci associated with a biotic interaction is an interesting approach that allows to correlate epigenetic changes with infection phenotypes [55]. In particular, the analysis of epigenetic regulation of *SlyWRKY75* provides a good model to search the role of introns as downstream gene regulatory elements which, in this case, include the Sly-miR1127-3p binding site. 

## 4. Materials and Methods

### 4.1. S. lycopersicum Growth Conditions

For each replicate, 40 tomato plants (*Solanum lycopersicum* cv. Ailsa Craig) were grown in commercial peat in a glasshouse with 16 h of daylight. Plants were infected after growing for 4 weeks (showing the clear development of third and fourth leaves). At least eight plants were chosen for both the control and infected conditions.

### 4.2. B. cinerea Bioassay

*Botrytis cinerea* strain B05.10 was kindly provided by Dr. Tudzynski (University of Munster, Germany). This strain was cultured on potato dextrose agar (PDA; Scharlau Microbiology 01-483-500) that contained 0.5% sucrose and was grown under dark conditions at 21 °C with exposure to UV-A light (350–400 nm) for 15 min every 3 h. Conidiospores were collected from the 7-day-old cultures with sterile water containing 0.02% (*v/v*) Tween-20, which was then filtered, quantified by a hemocytometer and adjusted to the appropriate concentration. Prior to infection, spores were resuspended in sterile water containing 25% (*v/v*) grape juice. The spore density for the infections was adjusted to 2.5 × 10^4^ pores/mL and was sprayed on the surface of all the leaves of at least eight plants. The control plants were sprayed in the same way, but without conidia. Mild disease symptoms were observed at 24 h, which was the collection time (Figure 1B). Growth of *B. cinerea* in the infected plants was determined as described by Crespo-Salvador et al. (2018) [28].

### 4.3. P. syringae Bioassay

*P. syringae* pv. tomato DC3000 was grown in King B medium at 28 °C with Rifampicin (50 mg mL^−1^) for 24 h. Bacterial suspensions were adjusted to 5 × 10^5^ cfu mL^−1^ in sterile MgSO4 (10 mM) with 0.01% of Silwet L-77 surfactant (Osi Specialties, Danbury, CT, USA). The third and fourth leaves of the tomato plants were challenged by dipping with a bacterial suspension, as described by Camañes et al. (2015) [22]. Disease was scored by bacterial DNA quantification (directly extracted or using the INPUT sample from the ChIP assay) or RNA. To do so, qPCR was performed using the primers for a constitutive gene of both *P. syringae* [56] and *S. lycopersicum*. Outer membrane protein F (OPRF) was used for bacteria and elongation factor 1 alpha (EF1a) for the plant. The bacterial gene signal was normalized with that of the plant (data are provided in Table S2).

### 4.4. Gene Expression Quantification by RT-qPCR

Total RNA extraction and cDNA generation were performed as described by Crespo-Salvador et al., (2018) [28]. Quantitative PCR was performed with the Tli RNase H Plus SYBR Premix Ex Taq (RR420W; TAKARA BIO INC., Kusatsu, Japan) in a LightCycler480 (Roche Diagnostics, Mannheim, Germany). Relative quantification was used to determine the differential gene expression. *S. lycopersicum SlyEF1a* expressions were used as internal standards. Real-time efficiency (*E*) was calculated for each primer pair according to the equation *E* = 10[−1/slope]. Relative expression was determined as described in Pfaffl (2001) [57]. Expression fold changes were expressed as infected/mock ratios (ΔΔ*C*t). Three replicates were performed with at least eight plants per genotype/condition with similar results. The normalized data are provided in Tables S1, S3 and S5. Novel primer sequences are shown in Table S8.

### 4.5. miRNA Assay

Extraction, quantification, and determination of the SlymiR11273p expression levels were performed as described by López-Galiano et al. (2018) [39]. The normalized data are provided in Table S6.

### 4.6. Chromatin Isolation and Immunoprecipitation Protocol

Both protocols are described by Crespo-Salvador et al. (2018) [28]. Briefly, for the chromatin isolation, *S. lycopersicum* leaves (2 g) were harvested and cross-linked with formaldehyde by the vacuum infiltration method [58]. Tissue was then frozen with liquid N_2_ and ground into fine powder. The chromatin was extracted following several centrifugation and cleaning steps [28] before being sonicated by a Bioruptor Sonication System Standard (Diagenode, UCD-200) at medium power in two 5-minute cycles (active for 30 s, inactive for 30 s) to obtain fragments of 100–1000 bp. Purified chromatin was then ready for immunoprecipitation.

Protein G-Dynabeads (10003D, Thermo Fisher Scientific, Waltham, MA, USA) were used along with chromatin to perform immunoprecipitation. Three main incubations were performed: first the pre-cleaning of both dynabeads and chromatin; second, the binding of antibodies with chromatin; third, the binding of the complexes formed in the previous incubation with dynabeads. Several cleaning steps with growing stringency buffers were performed to clean the sample of nonspecific binding particles. Finally, specifically bound chromatin was released from the dynabeads and its DNA was purified with the GeneJET PCR Purification Kit (K0702, Thermo Fisher Scientific, Waltham, MA, USA) for further qPCR analyses.

The employed antibodies were anti-H3K4me3 (07-473, Merck Millipore), anti-H3K9ac (07-352, Merck Millipore), anti-H3 (ab1791, abcam), and anti-RNAPII (ab817, abcam). The negative control was performed with no antibody.

The novel primer sequences are detailed in Table S6. The normalized data are provided in Tables S1 and S4 for *B. cinerea* and *P. syringae* infection, respectively.

### 4.7. Data Interpretation and Statistical Analysis

From the ChIP-qPCR signals, which resulted from each antibody and the INPUT samples, backgrounds were subtracted with their corresponding negative controls (no antibody), as described by Haring et al. (2007) [59]. The resulting signal of the H3K9ac and H3K4me3 antibodies was then normalized with the H3 antibody signal. The signal of the RNAPII antibody was directly normalized with the INPUT signal. The resulting signal was expressed as a % of H3 or INPUT immunoprecipitated. For all the comparisons, an *F* test (*p* < 0.05) was performed to determine the equality of variances of each infected and control condition combination. Consequently, a two-tailed Student’s *t* test (*p* < 0.05) was evaluated to determine the statistically significant difference between the mean values (± SE; *n* = 3). For the ratio comparisons, we normalized the data by transforming them into 10-base logarithms and then performing a one-tailed Student’s *t* test (*p* < 0.05), as shown in Table S3.

## Figures and Tables

**Table 1:** Results shown in a simplified and graphical way. The table includes significant changes in gene expression, histone marks, and RNAPII in the gene regions studied for the three biological replicates. In the ChIP experiments, changes of at least 4 times are indicated by ↑↑↑ or ↓↓↓ (positive and negative, respectively). Changes between 2 to 3.9 times are denoted by ↑↑ or ↓↓, and from 1.1 to 1.9 times are depicted by ↑ or ↓. Changes in gene expression of at least 100 times are represented by +++ or --- (positive and negative, respectively); differences from 10–99 times are depicted by ++ or --; and differences from 2 to 9 times are denoted by + or -. If no significant difference or a trend was found in all replicates, that field is empty.

Gene	Region		*B. cinerea*				*P. syringae*			
			Expression ^1^	H3K9ac	H3K4me3 ^1^	RNAPII	Expression	H3K9ac	H3K4me3	RNAPII
*SlyDES*	Promoter		**+++**	**↑↑↑**	**↑**	**↑↑↑**	**++**			
	TSS			**↑↑↑**	**↑↑**	**↑↑**		**↑**	**↑**	
	Exon 2 (end of CDS)			**↑↑↑**	**↑↑**	**↑↑↑**				
*SlyDOX1*	Promoter		**+++**	**↑↑**	**↑**	**↑**	**++**		**↑**	
	TSS			**↑↑**	**↑↑**	**↑**		**↑**	**↑**	
	Exon 1 (start of CDS)			**↑**	**↑↑**			**↑**	**↑**	
	Exon 10 (end of CDS)			**↑↑**	**↑↑**	**↑**		**↑**		
*SlyLoxD*	Promoter		**++**		**↑**	**↑↑**	**+**	**↑**		
	TSS					**↑↑**				**↑**
	Exon 1			**↑↑**		**↑↑**			**↑**	**↑**
	Exon 8 (3’-UTR)			**↑**		**↑**		**↑**		**↑↑**
*SlyWRKY75* ^2^	TSS		**++**	**↑↑**	**↑**	**↑↑**	**++**	**↑↑**	**↑↑**	**↑↑**
	Exon 1			**↑**	**↑↑**	**↑**		**↑↑**		**↑↑↑**
	Intron	miRNA binding region		**↑↑**		**↑↑**		**↑↑**	**↑**	**↑↑↑**
		Region 1		**↑↑**		**↑↑**		**↑↑**	**↑↑**	**↑↑↑**
		Region 2		**↑↑**	**↑↑**	**↑↑**		**↑↑↑**	**↑↑**	**↑↑↑**
		Region 3		**↑↑**	**↑↑**	**↑↑**		**↑↑↑**	**↑↑**	**↑↑↑**
	Exon 2 (3’-UTR)				**↑**			**↑↑**	**↑**	**↑↑↑**
miR1127-3p ^2^	Promoter		**−**	**↑**	**↓**					
	TSS				**↓**					
	3’ gene and intergenic region									

^1^ Data from [28] ^2^ Data from [39].

## Supplementary Materials

The following are available online at https://www.mdpi.com/2223-7747/9/3/300/s1, Table S1: ChIP *B. cinerea*, Table S2: gene expression, Table S3: ratio comparisons, Table S4: *P. syringae* quantitation, Table S5: ChIP *P. syringae*, Table S6: miR1127-3 expression, Table S7: *SlyWRKY75* processing primers, Table S8: *SlyWRKY75* intron primers.
